# Can Google Searches Predict the Popularity and Harm of Psychoactive Agents?

**DOI:** 10.2196/jmir.4033

**Published:** 2016-02-25

**Authors:** Wojciech Jankowski, Marcin Hoffmann

**Affiliations:** ^1^ Faculty of Chemistry Adam Mickiewicz University in Poznan Poznan Poland; ^2^ BioInfoBank Institute Poznan Poland

**Keywords:** drugs, narcotics, Internet, psychoactive agents, forecasting, trends

## Abstract

**Background:**

Predicting the popularity of and harm caused by psychoactive agents is a serious problem that would be difficult to do by a single simple method. However, because of the growing number of drugs it is very important to provide a simple and fast tool for predicting some characteristics of these substances. We were inspired by the Google Flu Trends study on the activity of the influenza virus, which showed that influenza virus activity worldwide can be monitored based on queries entered into the Google search engine.

**Objective:**

Our aim was to propose a fast method for ranking the most popular and most harmful drugs based on easily available data gathered from the Internet.

**Methods:**

We used the Google search engine to acquire data for the ranking lists. Subsequently, using the resulting list and the frequency of hits for the respective psychoactive drugs combined with the word “harm” or “harmful”, we estimated quickly how much harm is associated with each drug.

**Results:**

We ranked the most popular and harmful psychoactive drugs. As we conducted the research over a period of several months, we noted that the relative popularity indexes tended to change depending on when we obtained them. This suggests that the data may be useful in monitoring changes over time in the use of each of these psychoactive agents.

**Conclusions:**

Our data correlate well with the results from a multicriteria decision analysis of drug harms in the United Kingdom. We showed that Google search data can be a valuable source of information to assess the popularity of and harm caused by psychoactive agents and may help in monitoring drug use trends.

## Introduction

Misuse of psychoactive drugs [1] is one of the most serious social issues [2]. Illegal as they may be, they are readily available on the black market. Exposure to psychoactive drugs in some people may lead to addiction [3], affect the human brain [4], and modify human behavior [5], mood [6], and perception of the outside world. They can also cause death [7].

Harm caused by drug misuse is not easy to measure. The harmfulness of drugs may be approximated based on official statistics on crime, health, deaths, and social problems. On the other hand, questionnaires have been a valuable source of information for determining how harmful or dangerous the use of certain psychoactive drugs is. It is important to select respondents from those in contact with users of psychoactive agents and specialists in psychology, sociology, addiction therapy, and toxicology. The results of such studies are discussed for example by Nutt et al [8], who determined the harm of such agents using questionnaires distributed among a group of members of the Independent Scientific Committee on Drugs (subsequently renamed DrugScience) in the United Kingdom. They evaluated the agents on a 100-point scale with weighted criteria to include their relative importance. Several other papers and books have addressed the issue of harmful effects and popularity of the agents. Certain reports concluded that when drugs were used sporadically, cognitive functions were largely unaffected [9], while others showed that people who overuse such agents did not realize how harmful they were [10]. It was also confirmed that a method for responding to trends in drug use may be established based on multiyear studies [11]. Relevant books provide information not only about trends in drug use [12], but also about groups of people with a higher tendency to use drugs (by sex, age, or plans for the future) [13].

Laboratory studies of the harmful effects of drugs, most frequently animal studies, may be problematic because they are quite expensive and it is not clear how to translate the results to humans, the actual target. The major issue, however, is the number of agents that may have an impact on the human nervous system and its functions. With the development of chemical tests [14], a significant number of agents with effects similar to those of psychoactive drugs can be produced but have yet to be tested; therefore, they can be legally available even in countries with very restrictive antidrug laws. Designer drugs (novel psychoactive substance) [15,16] are a good example. The United Nations Office on Drugs and Crime (UNODC) [17] and the European Union [18] define a novel psychoactive substance as a new narcotic or psychotropic drug that is not scheduled under the Single Convention on Narcotic Drugs of 1961 or the Convention on Psychotropic Substances of 1971. NPSs may pose a public health threat comparable with that posed by substances listed in those conventions. They tend to be legally available because their ingredients are not included in the lists of illegal substances.

This study was inspired by a study based on Google Flu Trends [19], an investigation into influenza virus activity that showed that the number of persons with influenza worldwide can be monitored using the Google search engine. It was shown that the number of Internet search hits had 95% correlation with the number of persons who actually had influenza. Most important was that influenza virus activity can be determined quickly and updated daily, whereas specialized monitoring agencies provide weekly results based on data acquired with a certain delay.

All of these points led us to use the commonly available Google search engine to predict the popularity and harm of psychoactive agents. Here we describe our methods for obtaining popularity and harm rankings of drugs.

## Methods

Based on the report of Nutt et al [8] we decided to study 16 drugs: alcohol, amphetamine, benzodiazepines, buprenorphine, butane, cannabis, cocaine, ecstasy, gamma-hydroxybutyric acid (GHB), heroin, ketamine, khat, lysergic acid diethylamide (LSD), mephedrone, methadone, and methamphetamine. We choose the same substances as Nutt et al because we wanted to compare our results.

Our preliminary results showed a limitation of Google search engine, namely that for substances defined by 2 or more words the frequency of hits was either very small or very high, so we excluded these substances (anabolic steroids and crack cocaine). We also excluded tobacco and mushrooms because those keywords gave results that did not correspond to the subject of this research (information on the biological species rather than the drug substances). We entered the name of each drug between quote marks in Google (so that the exact string was searched for) with the SafeSearch filter off. Initially we considered including colloquial drug names in the list, but these could have had a negative impact on the results, because colloquial names often refer to concepts not related to psychoactive drugs in any way (eg, cocaine is called “snow”, while amphetamine is “speed”). Finally, we obtained the frequency of hits (N_i_) for the respective drugs. The relative popularity index for each drug (P_i_) was calculated as follows: P_i_=(N_i_/max(N_i_))×100% (1), where max(N_i_) is the drug with the highest frequency of hits (highest number of websites where that keyword is found).

Using the Google search engine with advanced search options may limit the results to websites that were available before a specific date. To generate the harmful effect ranking list, we again entered the agents into Google, but together with the terms “harmful” or “harm” (eg, “alcohol” “harm” OR “harmful”). The OR operator let us determine the frequency of page hits for the drug names *and* “harmful” or “harm” (N_i, harm_). Subsequently, we calculated harm indexes (H_i_) for the respective drugs as follows: H_i_=(N_i harm_/N_i_)×100% (2).

## Results

Table 1 shows the frequency of hits obtained in the Google search and the resulting relative popularity indexes calculated based on equation 1.

**Table 1:** Frequency of Google search hits for drugs (N_i_) and their relative popularity index (P_i_)^a^, June 20, 2014.

Drug no. (i)	Drug	Frequency of hits (N_i_)	Popularity index, % (P_i_)
1	Alcohol	389,000,000	100
2	Cannabis	59,100,000	15.2
3	Cocaine	58,600,000	15.1
4	LSD^b^	48,600,000	12.5
5	Heroin	46,800,000	12.0
6	Ecstasy	42,900,000	11.0
7	GHB^c^	23,500,000	6.0
8	Methadone	13,400,000	3.4
9	Butane	11,800,000	3.0
10	Khat	10,600,000	2.7
11	Amphetamine	9,070,000	2.3
12	Methamphetamine	8,780,000	2.3
13	Ketamine	8,400,000	2.2
14	Buprenorphine	6,400,000	1.6
15	Benzodiazepines	4,520,000	1.2
16	Mephedrone	2,050,000	0.5

^a^The relative popularity index was calculated as follows: P_i_=(N_i_/max(N_i_))×100%, where max(N_i_) is the drug with the highest frequency of hits.

^b^LSD: lysergic acid diethylamide.

^c^GHB: gamma-hydroxybutyric acid.

Table 1 shows that alcohol was the most popular psychoactive agent with a relative popularity index of 100%, followed by cannabis, 15.2%; cocaine, 15.1%; LSD, 12.5%; heroin, 12.0; ecstasy, 11.0%; GHB, 6.0%; methadone, 3.4%; butane, 3.0%; khat, 2.7%; amphetamine, 2.3%; methamphetamine, 2.3%; ketamine, 2.2%; buprenorphine, 1.6%; benzodiazepines, 1.2%; and mephedrone, 0.5%. It is not surprising in our ranking that alcohol is in first place because similar insights were reported in many papers [20-22] and reports [23,24]. The results change practically every day; therefore, the relative popularity index can be easily updated. It is an easy and fast method for data acquisition; only Internet access is needed.

The popularity indexes we obtained are similar to data from the UNODC *World Drug Report* from 2011 [25]. The UNODC report also documents the number of drug seizures. Most seized drugs were in the amphetamine-type stimulants group, followed by cannabis, cocaine, heroin, and morphine (last 2 are grouped and considered together). Our popularity ranking correlates with the UNODC report data: if we combine the amphetamine-type stimulants we looked at (ecstasy, amphetamine, and methamphetamine) in our ranking, this group is the most popular. Similar to the UNODC report, after amphetamine-type stimulants, the most popular drugs in our ranking were cannabis, cocaine, LSD and heroin.

Popularity indexes as calculated with equation (1) for illegal drugs are similar to those reported in the *European Drug Report 2014: Trends and Developments* [26], which uses the number of seizures of a drug as an indicator of its popularity. This could be a useful proxy, but it also depends on policy changes or the ease of hiding a drug (eg, LSD vs cannabis). Nevertheless, the report shows that the most frequently seized illegal drug is cannabis, second is cocaine, third is heroin, fourth is ecstasy, and then amphetamine, methamphetamine, and LSD. This list is quite similar to our ranking except for LSD, which has a higher popularity index than would be indicated by the number of seizures.

Changes in the frequency of hits for respective agents could be monitored practically daily, making it possible to follow drug use trends. We checked how relative popularity indexes change with the date when results were gathered. We compared data obtained on June 20, 2014 with data available before May 1, 2012, October 1, 2012, January 1, 2013, July 1, 2013, and February 1, 2014. Table 2 shows the resulting relative popularity indexes on different dates.

**Table 2:** Variation over time of relative popularity indexes (%) for drugs found by Google search, by date.

		May 1, 2012	October 1, 2012	January 1, 2013	July 1, 2013	February 1, 2014	June 20, 2014
max(N_i_)^a^		42,600,000	55,800,000	61,900,000	68,400,000	85,600,000	389,000,000
**Drug**							
	Alcohol	100	100	100	100	100	100
	Cannabis	6.3	5.9	6.9	7.7	10.1	15.2
	Cocaine	10.5	9.4	10.3	10.4	11.4	15.1
	LSD^b^	6.4	6.8	7.9	8.8	8.8	12.5
	Heroin	8.8	9.1	10.0	10.4	11.0	12.0
	Ecstasy	6.2	5.1	5.6	6.1	6.6	11.0
	GHB^c^	0.9	1.1	1.3	1.5	1.7	6.0
	Methadone	1.8	2.1	2.2	2.0	1.7	3.4
	Butane	1.8	2.1	2.3	2.3	2.2	3.0
	Khat	1.3	1.2	1.3	1.4	1.6	2.7
	Amphetamine	1.2	1.5	2.2	1.6	1.6	2.3
	Methamphetamine	1.5	1.6	1.6	1.6	1.6	2.3
	Ketamine	2.6	2.3	2.2	1.9	1.6	2.2
	Buprenorphine	1.0	0.6	0.6	0.6	0.5	1.6
	Benzodiazepines	1.7	1.3	1.3	1.2	1.0	1.2
	Mephedrone	0.1	0.1	0.1	0.1	0.1	0.5

^a^max(N_i_): drug with the highest frequency of hits (highest number of websites where that keyword is found).

^b^LSD: lysergic acid diethylamide.

^c^GHB: gamma-hydroxybutyric acid.

The most popular psychoactive agent was alcohol on all the studied days. As Table 2 shows, the popularity indexes of heroin, cocaine, cannabis, GHB, ecstasy, and LSD all rose greatly with respect to alcohol over the last 2 years. Changes in popularity of other drugs were not as great, but some of them switched places in the ranking. These data show that between May 1, 2012 and June 20, 2014 cannabis became more popular than cocaine and heroin became less popular than LSD. Similar results are also shown in the UNODC’s *World Drug Report* [25]. The *European Drug Report 2014* [26] also showed that heroin become less popular and the number of seizures declined. This report also showed that the quantity of seized cannabis increased every year while that of seized cocaine decreased. Because the Internet data can be captured so fast, it is possible to monitor or maybe even respond to changes in drug popularity, making it possible to identify what psychoactive agents come into fashion and to respond to new trends. Moreover, on the basis of changes in popularity, we can point to which substances may be being used together. According to Table 2, we can assume that many users of different substances may also use cannabis. Changes in the popularity of this drug correlate with changes in the popularity of cocaine, LSD, heroin, ecstasy, khat, GHB, and methamphetamine. Changes in the popularity of cocaine correlate with changes in the popularity of ecstasy, khat, and GHB. Changes in the popularity of ecstasy correlate with changes in the popularity of GHB, khat, and mephedrone. Similarly, changes in the popularity of GHB correlate with changes in popularity of khat, methamphetamine, and mephedrone; methadone with methamphetamine and mephedrone; khat with methamphetamine and mephedrone; and methamphetamine with mephedrone. On the basis of these results, we can assume that these groups of substances may have been used together.

Table 3 presents data assessing how harmful a given drug seems to be based on the crude methods described in the Methods sections.

**Table 3:** Harmfulness of drugs as assessed by harm index (H_i_)^a^ (data acquired from Google search on June 20, 2014) and their harm score.

Drug no. (i)	Drug	N_i, harm_^b^	Harm index, % (H_i_)	Harm score^c^
1	Alcohol	107,000,000	27.5	72
2	Cocaine	15,000,000	25.6	27
3	Heroin	11,700,000	25.0	55
4	Benzodiazepines	467,000	10.3	15
5	Khat	711,000	6.7	9
6	Buprenorphine	374,000	5.8	7
7	Methamphetamine	513,000	5.8	33
8	Amphetamine	465,000	5.1	23
9	Ketamine	411,000	4.9	15
10	Cannabis	2710,000	4.6	20
11	Mephedrone	92,000	4.5	13
12	Methadone	543,000	4.1	14
13	Butane	386,000	3.3	11
14	Ecstasy	669,000	1.6	9
15	GHB^d^	291,000	1.2	19
16	LSD^e^	580,000	1.2	7

^a^The harm index was calculated as follows: H_i_=(N_i harm_/N_i_)×100%, where N_i_ is the frequency of hits for the drug.

^b^N_i harm_ is the frequency of page hits for the drug names *and* the search terms “harmful” OR “harm”.

^c^Data from Nutt et al [8].

^d^GHB: gamma-hydroxybutyric acid.

^e^LSD: lysergic acid diethylamide.

Among the studied substances, alcohol had the highest harm index (27.5%), followed by cocaine (25.6%), heroin (25.0%), benzodiazepines (10.3%), khat (6.7%), buprenorphine (5.8%), methamphetamine (5.8%), amphetamine (5.1%), ketamine (4.9%), cannabis (4.6%), mephedrone (4.5%), methadone (4.1%), butane (3.3%) ecstasy (1.6%), GHB (1.2%), LSD (1.2%). We compared our results for the drugs with the results obtained in another study (Nutt et al [8]) in which a different harm ranking list [8] was suggested, with a harm score assigned to agents. They calculated the harm index as a percentage, with the harm score having no unit. Nutt at al [8] assigned 100 points to a theoretical most-harmful substance, which has not been discovered yet. Similar to our ranking, their highest harm score was for alcohol. However, their second-ranking drug was heroin, whereas in our ranking cocaine has the second-highest harm score. The harm score for cocaine is smaller than that for methamphetamine, whereas methamphetamine’s harm index is about 5 times smaller than that for cocaine. Similarly, amphetamine, cannabis, ketamine, benzodiazepines, and GHB have harm scores similar to that for cocaine, but their harm indexes are 5 or 6 times smaller than the harm index for cocaine. Both rankings (except alcohol) also have ketamine, mephedrone, ecstasy, and LSD in the same positions, but in all cases the harm score is bigger than the harm index.

**Figure 1:**
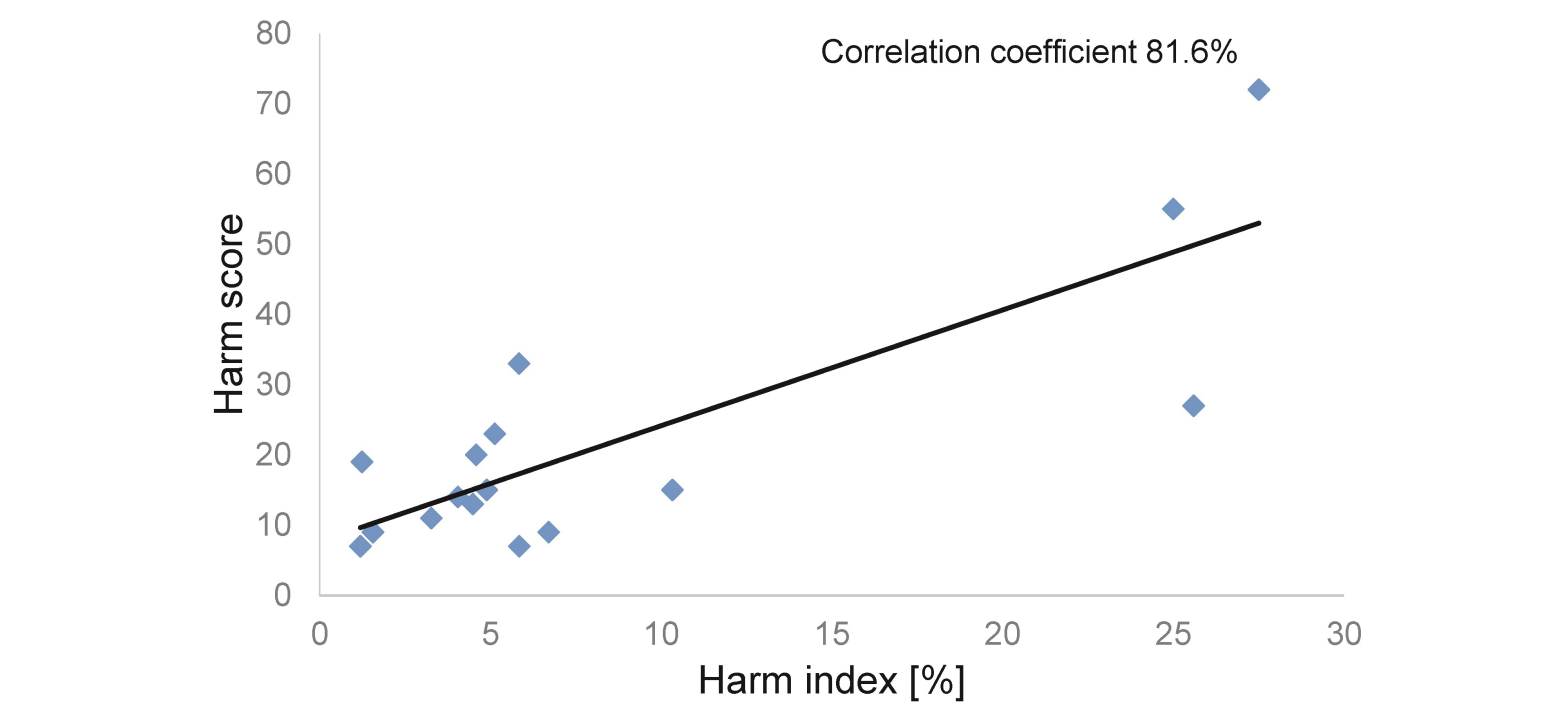
Correlation between harm scores obtained by Nutt et al [8] and the proposed harm index for a series of drugs identified by Google search (blue squares).

We plotted harm scores from Nutt et al [8] and our harm indexes to visualize the correlation between our studies (Figure 1).

The correlation coefficient between the harm score ranking and the harm index was 81.6%. The calculated *P* value was 0.000143, showing a significant correlation, with the significance level set at 0.01. We should stress that the *P* value does not demonstrate that data from the Independent Scientific Committee on Drugs [8] and our Internet data are correlated because they both address the same topic; however, that they do correlate and the significance of the *P* value indicate that the correlation is unlikely to arise by chance. These results show that a Web search with simple numerical analysis of the results may provide valuable data in a much shorter time frame. The results from using popular names for drugs and the term “harm” as the indicator of adverse effects should, however, be assessed with particular caution, as harmful effects are much more complex. Nevertheless, this method may be viewed as an initial proxy for a concept as complex as harm (which includes social factors, health issues, and mental problems).

## Discussion

Information derived from the Internet should be assessed with particular caution and always verified against sound data from other sources. Google search data may be and indeed sometimes are misleading. The simplest example is the phrase “cat” OR “dog”. A Google search returns well over 2 billion webpages containing the word “cat” and well over 1 billion websites with the term “dog”. Shockingly, when we combine these 2 words with the OR operator, we obtain less than half a billion webpages. In this case the inclusive disjunction operator does not show results that include both sets.

Nevertheless, the Internet has become a mine of information, which, when verified with other sources of data, may be beneficial. Based on Google Web searches verified against data from the UNODC [25], the European Monitoring Centre for Drugs and Drug Addiction [26], and Nutt et al [8], we have shown that data from Web searches—when treated with caution—may be a valuable source of information on drug use. We obtained popularity and harm ranking lists for selected psychoactive drugs. Furthermore, we recorded changes in drug popularity at various time points over 2 years (2012-2014). The proposed approach may help indicate early changes in drug popularity and may be useful for preventing drug addiction. Interestingly, this crude and simple approach to estimating harm associated with a given drug harm index correlated very well (correlation coefficient 81.6%) with the much more sophisticated method proposed by Nutt et al [8] of calculating harm scores. Our results seem to indicate that, with respect to alcohol, the popularity of psychoactive agents has increased significantly over the 2 years 2012-2014, in particular the popularity of cannabis (from 6% to 15%), cocaine (from 10% to 15%), LSD (from 6% to 12%), and ecstasy (from 6% to 11%).

## Abbreviations

GHB: gamma-hydroxybutyric acid
LSD: lysergic acid diethylamide
UNODC: United Nations Office on Drugs and Crime
